# Reduction in initiations of drug-sensitive tuberculosis treatment in South Africa during the COVID-19 pandemic: Analysis of retrospective, facility-level data

**DOI:** 10.1371/journal.pgph.0000559

**Published:** 2022-10-11

**Authors:** Mariet Benade, Lawrence Long, Gesine Meyer-Rath, Jacqui Miot, Denise Evans, Jeanne-Marie Tucker, Harry Moultrie, Sydney Rosen

**Affiliations:** 1 Department of Global Health, Boston University School of Public Health, Boston, Massachusetts, United States of America; 2 Health Economics and Epidemiology Research Office, Department of Internal Medicine, School of Clinical Medicine, Faculty of Health Sciences, University of Witwatersrand, Johannesburg, South Africa; 3 Clinton Health Access Initiative, Johannesburg, South Africa; 4 Division of the National Health Laboratory Services, National Institute of Communicable Diseases, Johannesburg, South Africa; 5 School of Pathology, Faculty of Health Sciences, University of the Witwatersrand, Johannesburg, South Africa; University of Cape Town, SOUTH AFRICA

## Abstract

In response to the global pandemic of COVID-19, South Africa implemented a strict lockdown in March 2020 before its first COVID-19 wave started, gradually lifted restrictions between May and September 2020, and then re-imposed restrictions in December 2020 in response to its second wave. There is concern that COVID-19-related morbidity and mortality, the deprioritization of TB activities, fear of transmission, and societal restrictions led to a reduction in tuberculosis (TB) treatment initiations. We analysed monthly public sector, facility-level data from South Africa’s District Health Information System (DHIS) from January 2019 to April 2021 to quantify changes in TB treatment initiation numbers stratified by province, setting, and facility type and compared the timing of these changes to COVID-19 case numbers and government lockdown levels. At the 1189 facilities that reported observations for all 28 months of our study period, TB treatment initiations in 2020 were 20.4% lower than in 2019 and 21.9% lower in the first four months of 2021 than in 2019. At the 3669 facilities that reported observations in ≤28 months, numbers of TB treatment initiations declined sharply in all provinces in May-August 2020, compared to the same months in 2019. After recovering somewhat in the last four months of 2020, numbers plummeted again in early 2021. Percentage reductions were somewhat larger in urban and peri-urban areas than in rural areas. Most provinces experienced a clear inverse relationship between COVID-19 cases and TB treatment initiations but little relationship between TB treatment initiations and lockdown level. The COVID-19 pandemic and responses to it resulted in substantial declines in the number of individuals starting treatment for TB in South Africa and risked progress toward achieving TB management goals. Exceptional effort will be needed to sustain gains in combating TB.

## Introduction

The global pandemic of COVID-19 has disrupted many aspects of life for people around the world, including access to care for common diseases such as tuberculosis (TB). Modeling studies and preliminary data reports provided early warnings about how COVID-19-related illness, fear of COVID-19 transmission, and national and local measures to prevent transmission, such as stay-at-home orders, could harm timely access to TB testing and treatment [1–3]. Successfully initiating patients on TB treatment requires that both TB diagnostic and treatment services function properly, that household contacts and people with TB symptoms come forward for screening and diagnosis, and that those who test positive return to the healthcare facility to initiate TB treatment. There is reason for serious concern that global targets for TB treatment were not met in 2020 and may have been missed in 2021 [4, 5].

South Africa’s first wave of COVID-19 began in June 2020 and ended in August 2020; a second wave was recognized from December 2020 to January 2021 and a third wave began in June 2021, with provincial variations [6]. South Africa declared a national state of disaster on 15 March 2020 and implemented strict lockdown measures such as stay-at-home orders, travel restrictions, and the closure of schools, non-essential businesses, and most public transportation on 27 March 2020. Restrictions were gradually lifted in increments between 1 May and 21 September 2020 before being tightened again in late December 2020, in the face of a second wave of COVID-19 infections and then easing again starting in February 2021 [7, 8].

During the initial lockdown, individuals were permitted to leave their homes to seek healthcare, but public transportation was not readily available, and many primary healthcare clinics temporarily closed or reduced operating hours due to staff cases and exposures requiring quarantine, other staff absences, stockouts of supplies, and/or redeployment of resources for COVID-19 screening and testing [8, 9]. Patients may also have avoided visiting clinics due to fear of COVID-19 transmission [10]. Importantly, a large documented downturn in screening and testing for TB during the lockdowns likely reduced the number of candidates for TB treatment initiation [11].

Although a few articles and reports have discussed the potential impact of COVID-19 restrictions on healthcare service delivery in South Africa [8, 12], precise estimates of the decline in TB tests are available [11], and information about the TB care cascade from one province, KwaZulu Natal, are available [13], we could not locate any empirical studies that report changes in numbers of TB treatment initiations for the entire country, nor has anyone compared TB treatment initiation numbers to COVID-19 incidence and response stages. Unpublished reports, such as those of the WHO [14], Global Fund [15], and South African National Institute for Communicable Diseases [1], have generally been brief, aggregate descriptions, based on only very early pandemic data, or limited to TB diagnosis rather than treatment.

To provide a detailed understanding of the impact of the COVID-19 pandemic and pandemic control measures and improve future decision making, we used national data to quantify changes in TB treatment initiation rates in the South African national TB control program between January 2019 and April 2021 by province, to identify facility characteristics associated with changes, and to compare the timing of changes in TB treatment initiation numbers with the timing of COVID-19 infections and restrictions.

## Methods

### Data

The District Health Information System (DHIS) [16] is used by the South African Government to collect aggregate (facility-level) data about service delivery at the more than 4,000 public sector facilities in the country, which provide health services for roughly 84% of South Africa’s population [17]. It is estimated that between 85 and 96% of all first-line TB treatment is provided by the public sector in South Africa [18]. Facilities report monthly on a wide range of process indicators, including the number of individuals initiated on TB treatment. Reporting is usually fairly complete; although multi-month lags in data entry for individual facilities are common, backlogs are generally cleared in the subsequent months. The accuracy of the data reported has been shown to vary among sites [19].

We accessed monthly national DHIS data for 01 January 2019 to 30 April 2021 for all facilities in the DHIS data set as of July 2021. In the dataset, each facility reports the number of adults and children (above 5 years) initiated on TB treatment each month. We restricted our dataset to facilities that had at least one drug-sensitive (DS-TB) treatment initiation documented in 2019 and to primary healthcare clinics, community health centres, and hospitals serving the general population, the three levels of facilities that provide the vast majority of public sector healthcare in South Africa. We excluded non-traditional TB treatment sites such as mobile clinics, correctional facilities, specialised psychiatric hospitals, specialised TB hospitals, satellite clinics, specialised centres, and health posts. Data were censored as of 30 April 2021 to allow facilities up to three months to complete their DHIS entries before our data set was extracted in July 2021.

We noted that many facilities were missing observations (number of TB treatment initiations/month) for up to several months in the study period, including in 2019, which was our baseline (pre-COVID-19) comparison year. To compensate for this, we included in the analysis only observations for each month in 2020 and/or 2021 for each facility where we could match to an observation in the corresponding month in 2019. This matched data set is referred to as the “full data set.” (Monthly reports of 0 TB treatment initiations were common and not regarded as missing, but facilities that reported no initiations in any month in 2019 were excluded.)

Since this methodology led to the full data set containing a different number of facilities in each calendar month, we also created a “limited data set” containing only facilities that reported data for every month in the study period. In the limited data set, each monthly observation represented the same set of facilities, holding the number of facilities constant and allowing us to compare absolute numbers of TB treatment initiations over time, rather than just percentage changes.

We also collected information from the original DHIS data set on the province, setting (urban, peri-urban, or rural), and level (clinic, hospital, or community health centre) for each facility [20]. In addition to DHIS data, we used publicly-available data on monthly COVID-19 cases in each province and on the dates at which different levels of lockdown were imposed [6].

DHIS data were accessed under a Data Users Agreement approved by the Chief Director: Health Information Research Monitoring & Evaluation of the National Department of Health of South Africa for purposes of this analysis. As all data were aggregate, facility-level indicators and no human subjects data were used, no research ethics review was required. Additional information regarding the ethical, cultural, and scientific considerations specific to inclusivity in global research is included S1 Checklist.

### Analysis

Using the limited data set, we first estimated absolute differences in numbers of drug-sensitive TB treatment initiations over time from January 2019 to April 2021. Next, using the full data set, we calculated the total number of TB treatment initiations per study month and year for in 2019 and percentage changes from the 2019 baseline in 2020 and 2021 for each calendar month. For example, the number of initiations in March 2019 was compared to that in March 2020 and March 2021, to control for seasonal differences in healthcare utilization unrelated to COVID-19. We also stratified the full data set by province, facility type, and setting and compared changes in TB treatment initiation numbers between the groups to determine if specific subsets of facilities experienced larger or smaller changes in service volumes. We also examined the relationships over time among TB treatment initiations, COVID cases, and lockdown levels. Finally, we estimated the unmet need for TB treatment initiations nationally by multiplying the percentage reduction in TB treatment initiations in the limited dataset by the total number of TB treatment initiations nationally in 2019.

## Results

### Study population

Of the 4,133 healthcare facilities included in the original data set, we excluded 464 facilities that were non-traditional TB treatment sites (Table 1). The full analytic data set included 89% (3,669/4,133) of all facilities in the original data set. S1 Table provides the number of facilities included in and excluded from the full data set in each month in the observation period. It should be noted that although only 89% of facilities were included, 97% of all TB treatment initiations were carried out at these sites in 2019. The limited data set, which was restricted to facilities that reported observations for every month of the study period, included just over a quarter (29%, 1,189 of 4,133 facilities) of the original DHIS data set.

**Table 1:** Characteristics of facilities included in the full and limited data sets.

Characteristic	Original DHIS data set		Full analytic data set		Limited analytic data set	
	**Number of facilities (%)**	**Number TB treatment initiations in 2019 (%)**	**Number of facilities (%)**	**Number TB treatment initiations in 2019 (%)**	**Number of facilities (%)**	**Number TB treatment initiations in 2019 (%)**
Number of facilities	4,133 (100)	157,327 (100)	3,669 (89)	152,122 (97)	1,189 (29)	106,409 (68)
Facility type*						
Primary healthcare clinics	3044 (74)	85,512 (55)	3,044 (83)	83,115 (55)	841 (71)	51,705 (49)
Community health centres	332 (8)	27,494 (18)	332 (9)	27,381 (18)	182 (15)	23,308 (22)
Hospitals	293 (7)	41,842 (27)	293 (8)	41,626 (27)	166 (14)	31,396 (29)
Other	464 (11)	2,479	0	n.a.	0	n.a.
*Setting*						
Urban	1,816 (44)	109,295 (70)	1614 (44)	105,774 (70)	817 (69)	82,860 (78)
Peri-urban	405 (10)	13,158(8)	325 (9)	12,482 (8)	111 (9)	8,547 (8)
Rural	1,912 (46)	34,874 (22)	1730 (47)	33,866 (22)	261 (22)	15,002 (14)
*Province*						
Eastern Cape	890 (22)	31,965 (20)	836 (23)	30,932 (20)	282 (24)	22,197 (21)
Free State	285 (7)	8,725 (6)	246 (7)	8,487 (6)	71 (6)	4,666 (4)
Gauteng	423 (10)	22,342 (14)	391 (11)	21,907 (14)	190 (16)	15,896 (15)
KwaZulu Natal	786 (19)	42,365 (27)	664 (18)	40,990 (27)	263 (22)	32,813 (31)
Limpopo	497 (12)	6,981 (4)	472 (13)	6,831 (4)	43 (4)	2,912 (3)
Mpumalanga	321 (8)	7,339 (5)	312 (9)	7,104 (5)	48 (4)	2,230 (2)
Northern Cape	204 (5)	9,890 (6)	161 (4)	9,595 (6)	32 (3)	1,844 (2)
North West	341 (8)	5,272 (3)	309 (8)	4,829 (3)	71 (6)	4,786 (5)
Western Cape	386 (9)	22,448 (14)	278 (7)	21,447 (14)	189 (16)	19,065 (18)

*Public sector sites only

### Changes in TB treatment initiations compared to 2019

**Fig 1:**
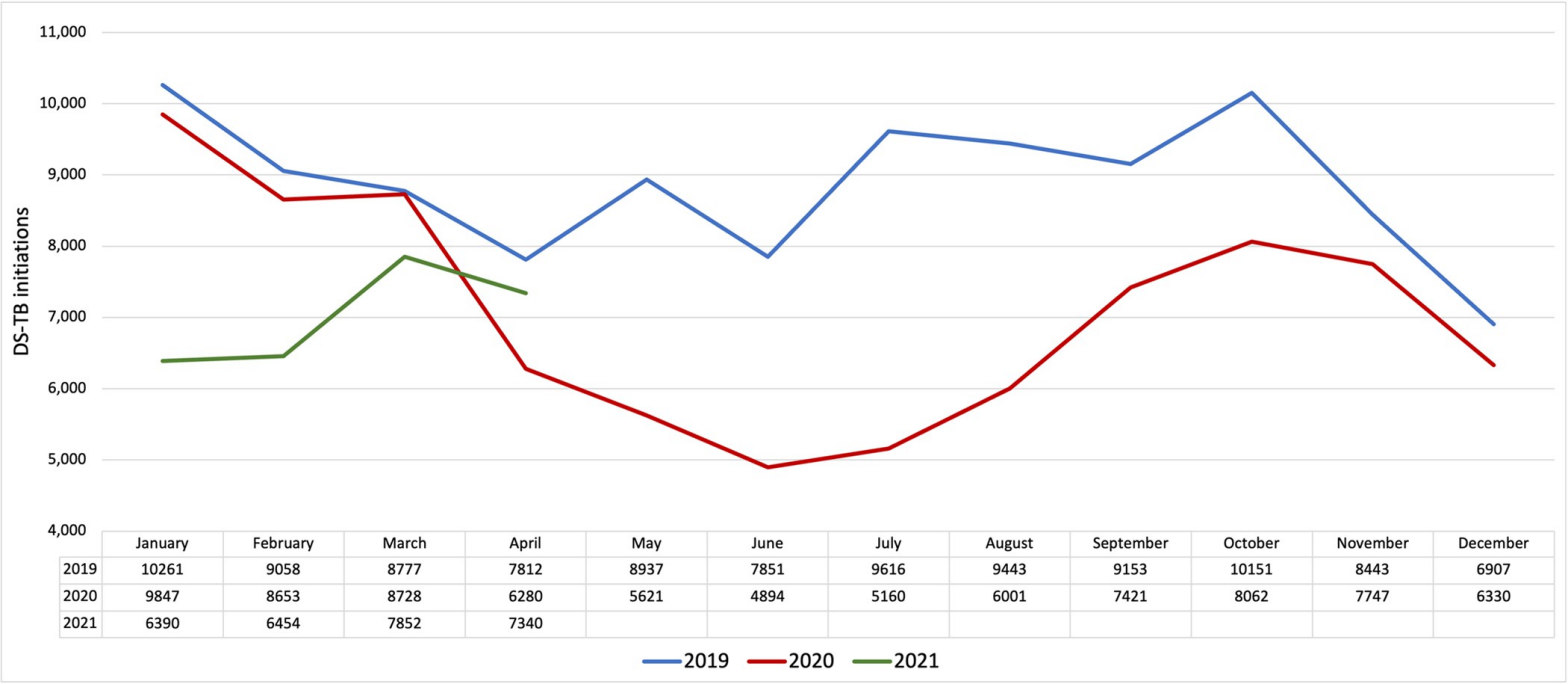
TB treatment initiations among facilities in limited dataset (N = 1,189), January 2019-April 2021.

**Fig 2:**
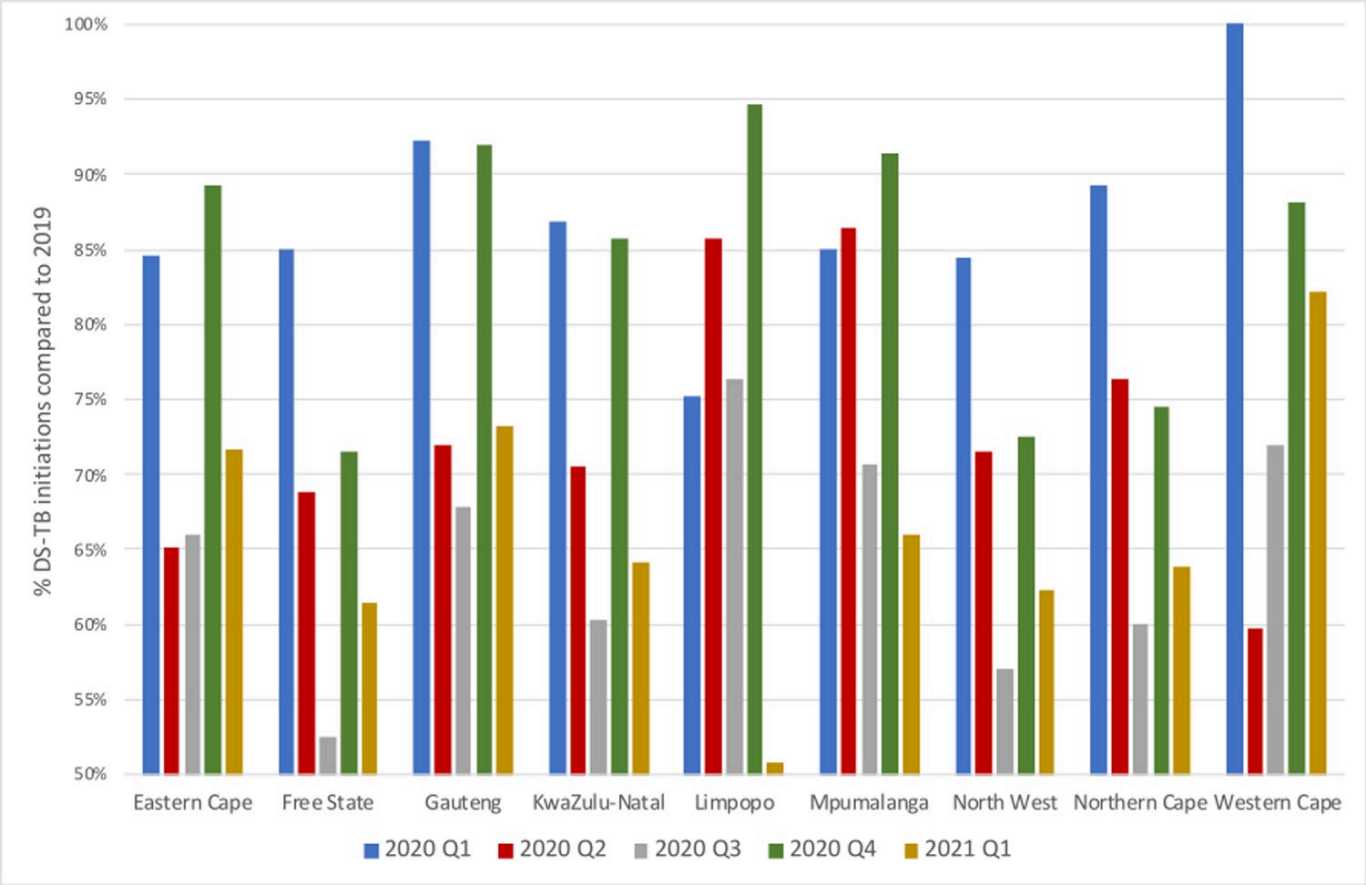
TB treatment initiations in 2020 as a percentage of the number of initiations in 2019, by province.

Fig 1 illustrates the total number of patients initiated on TB treatment per month over the full study period at facilities in the limited data set. As explained above, comparisons of absolute numbers of initiations in the full data set can only be done month by month, as each monthly total in the full data set represents a different number of facilities. For the full data set, Fig 2 presents the number of TB treatment initiations in calendar year 2020 and the first quarter of 2021 in each province of South Africa as a percentage of the number of initiations in each quarter of 2019.

At the national level, the number of patients initiated on TB treatment in the limited data set was similar in January-March 2019 and 2020 (Fig 1). There was then a steep decline in April-June 2020, compared to the same months in 2019. This discrepancy persisted until November 2020, with some recovery in November-December 2020 and again in April 2021, between waves of COVID-19 infection. At the facilities included in the limited data set, which comprise 29% of facilities in the original DHIS data set, a total of 21,665 fewer patients were initiated on TB treatment in 2020 than in 2019, or a reduction of 22%. Extrapolated to the entire population, this would indicate an unmet need of 34,490 people not initiated on TB treatment in 2020.

In the much larger full data set, representing 89% of facilities and 97% of TB treatment initiations in 2019, all but three provinces experienced a sharp drop-off between the first and second quarters of 2020 (Fig 2). Major declines came the following quarter in Mpumalanga and the Northern Cape. Limpopo only saw dramatic declines in treatment initiation during the second wave, though it should be noted that it has a small TB program in comparison with other provinces. After that, while patterns were roughly consistent across the provinces, the timing and magnitude of the changes differed, with the fastest declines and earliest recoveries in the Western Cape and Gauteng. All provinces experienced some recovery in the fourth quarter of 2020 before falling off again in early 2021.

**Fig 3:**
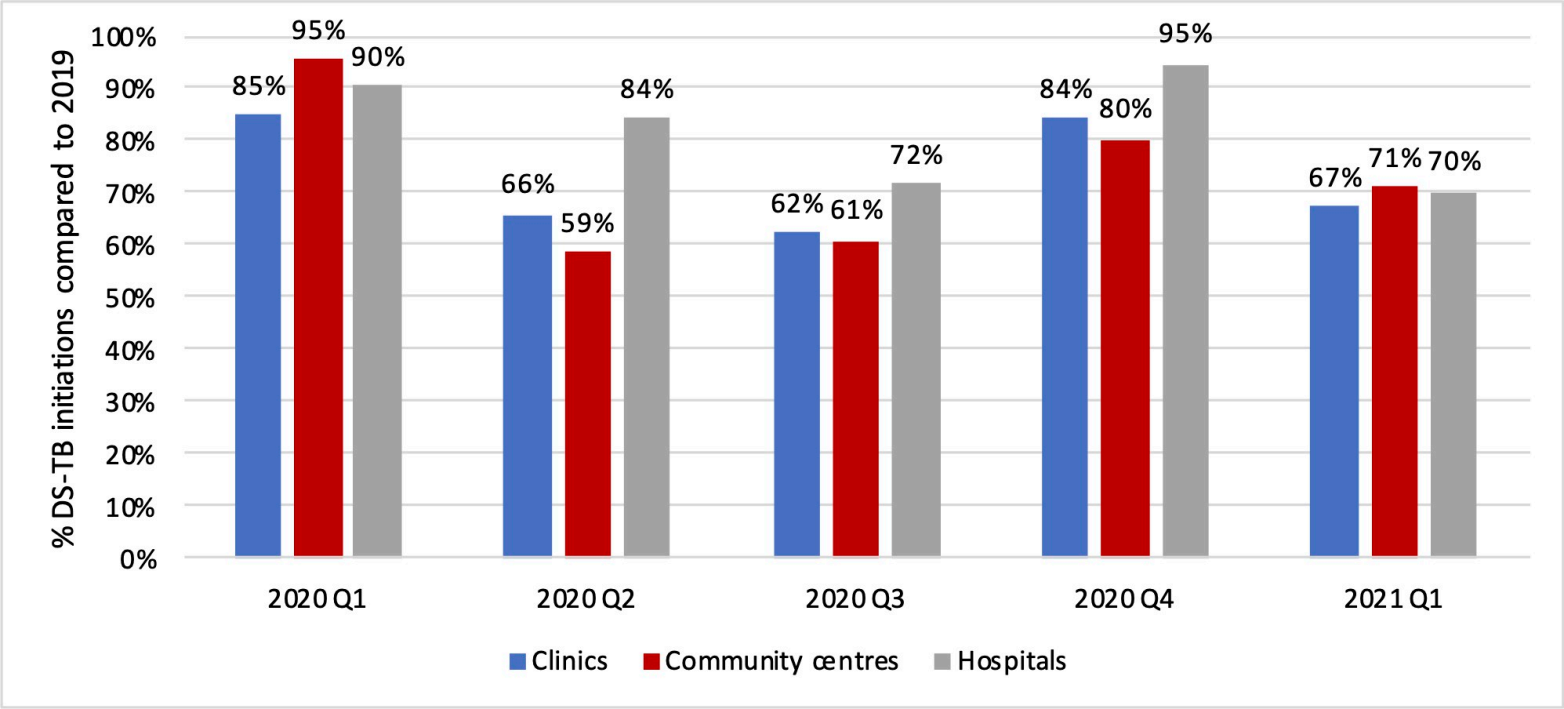
Drug sensitive TB initiations as proportion of initiations in the same quarter of 2019, by facility level.

**Fig 4:**
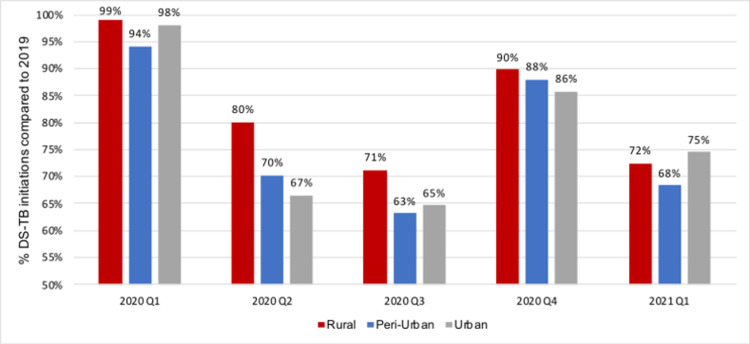
TB treatment initiations as proportion of initiations in same quarter of 2019, by setting.

We next looked at the changes in TB treatment initiation numbers by facility level and setting in the full data set, as shown in Figs 3 and 4.

Reductions in DS-TB treatment initiation numbers were lower in percentage terms in hospitals (compared to primary health clinics and community health centres) and rural areas (compared to urban and peri-urban settings). Initiations at primary health clinics, which are responsible for 55% of all initiations in the country, experienced a decrease of more than 30% in the second (6,609) and third (8,383) quarters of 2020, while community health centres saw decreases of 40% (Q2: 2597; Q3: 2867) over the same time period. Facilities in rural, peri-urban, and urban areas all experienced losses in the second and third quarter of 2020 and recovered to some extent in the last quarter of the year. Facilities in rural areas seemed to be affected least throughout 2020, and facilities in urban areas most.

### Changes in TB treatment initiation numbers, COVID-19 cases, and lockdown levels

**Fig 5:**
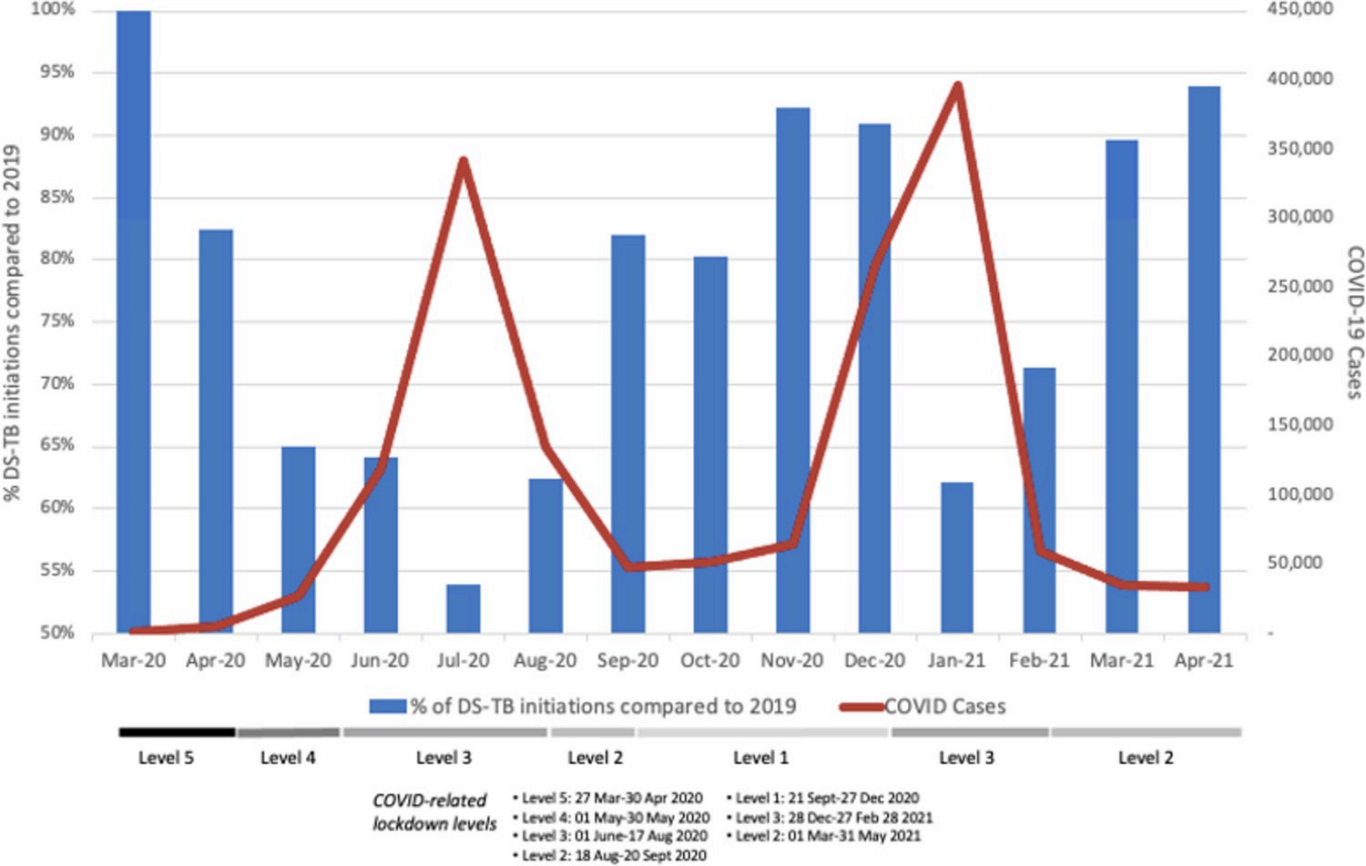
Proportion TB treatment initiations compared to the same month in 2019 and new COVID-19 cases per month, March 2020-April 2021.

**Fig 6:**
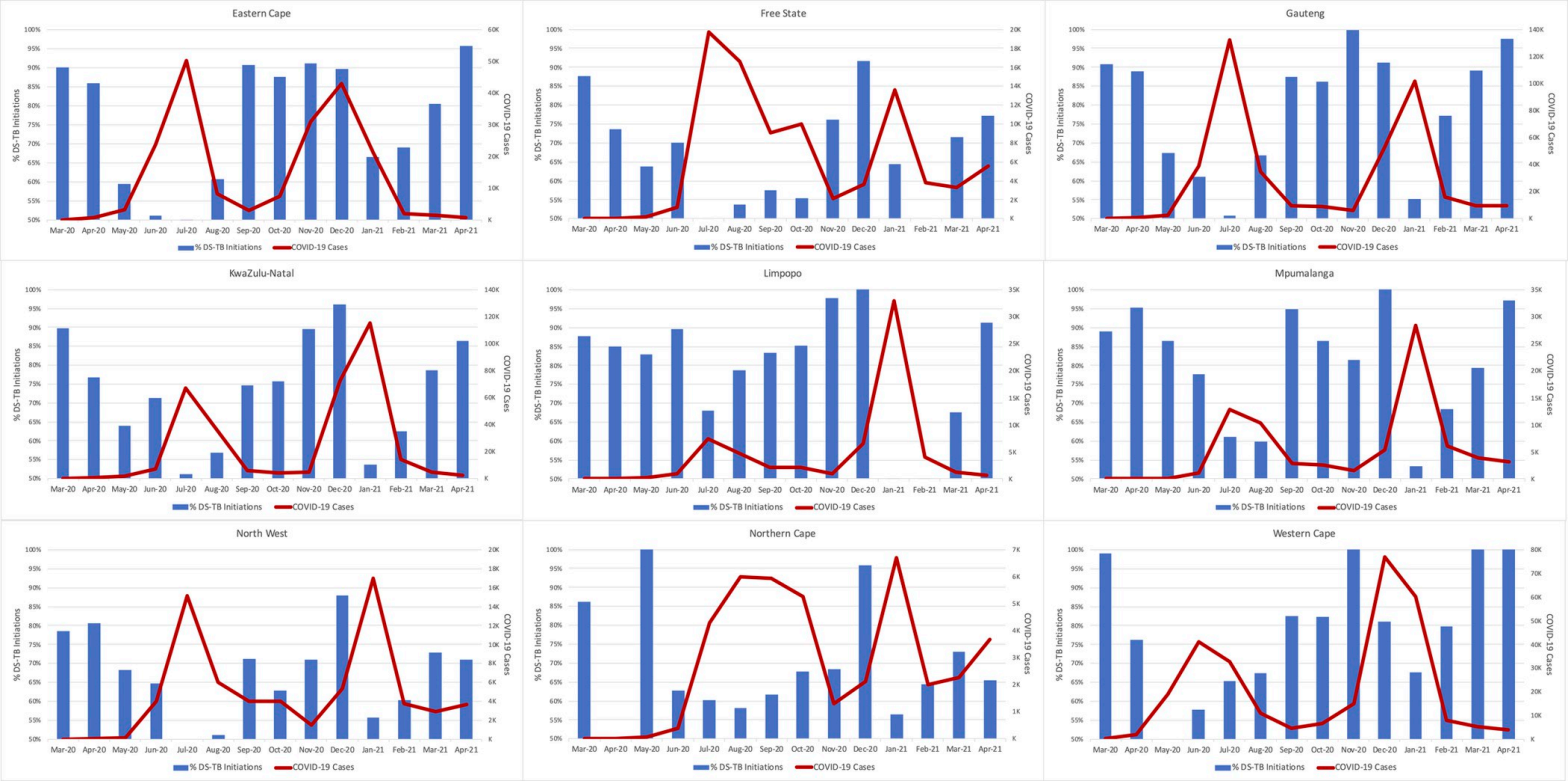
Proportion TB treatment initiations compared to the same month in 2019 and new COVID-19 cases per month, March 2020-April 2021, by province.

Finally, we compared the timing of TB treatment initiations, COVID-19 cases, and national lockdown restrictions. Fig 5 presents these variables for the country as a whole and Fig 6 for each province, using the full data set.

In most provinces, a decline in TB treatment initiations was seen during the level 5 lockdown in late March 2020 and April 2020. Diagnosed cases of COVID-19 began to rise steeply only in May 2020, after the shift to lockdown level 4, which eased some restrictions. After the initial decline, most provinces experienced a clear inverse relationship between COVID-19 cases and TB treatment initiations but little relationship between TB treatment initiations and lockdown level. The pattern of TB treatment initiations and COVID-19 cases in the Northern Cape Province is somewhat anomalous; the data do not allow us to explain the reason for this outlier, which is South Africa’s least populous province.

## Discussion

In this analysis of aggregate data reported by 89% of public sector healthcare facilities in South Africa, we found that the number of individuals initiating TB treatment fell by more than one fifth between 2019 and 2020. TB treatment initiations declined in all provinces and for all facility types, sizes, and settings. Although there was some recovery between the first and second COVID-19 waves, numbers remained well below those seen in 2019. After the initial drop-off in April-May 2020, which preceded South Africa’s first wave of COVID-19, TB treatment initiations generally fell as diagnosed COVID-19 infections rose, rather than in direct proportion to the stringency of the national lockdown.

The findings we report here are of global relevance, because South Africa remains one of the world’s eight highest burden TB countries, with among the highest TB incidence in the world. The disruption in TB diagnosis and treatment associated with the COVID-19 pandemic thus threatens not only South Africa’s own efforts to reduce TB morbidity and mortality, but contributes to the global reversal in progress against TB [21].

The limited data set used in this analysis covered 29% of South African public sector facilities. If the overall relative reduction in TB treatment initiations calculated for the limited data set is extrapolated to 100% of the public sector facilities in the country, we estimate that some 35,000 fewer TB treatment initiations took place in 2020 than in 2019. We do not know how much of this discrepancy is due to the large and early declines reported in numbers of TB tests conducted [1, 13] which would have reduced the number of known TB-positive patients who could potentially have started TB treatment, and how much to barriers to access to care related to COVID-19 and the responses to it. (We also considered whether behavioural changes early in the pandemic, such as social distancing and the wearing of face masks, suppressed TB transmission enough to reduce later disease incidence and thus limit the number of patients in need of TB treatment. Given the usual time interval of 2 years between exposure and development of symptoms [22], however, we doubt that any effect of reduced transmission after March 2020 would be apparent in our data set ending in April 2021.)

Regardless of its causes, the impact of the “missing” TB treatment initiations on TB-related morbidity and mortality rates will depend on whether and when undiagnosed people with TB come forward for TB diagnosis and treatment initiation. Most likely, some patients will initiate TB treatment relatively soon after they otherwise would have and will suffer few consequences as a result of the delay. Others, however, will become more seriously ill than they otherwise would have been and/or will incur higher medical care costs, and some will likely die of TB. Delays in starting TB treatment will also almost certainly increase TB transmission, a setback to the country’s efforts to achieve national and global goals for TB management [23, 24].

Other literature on the impact of the COVID-19 pandemic on TB treatment initiation supports our findings, although none reports these findings by province, setting, or facility type. The most relevant, Arsenault et al (2022) [13], reporting results from KwaZulu-Natal Province only, found a 25% reduction in TB treatment initiation between the 15 months preceding the WHO declaration of COVID-19 as a pandemic and the first 9 months after. As each of South Africa’s nine provinces has jurisdiction over health care service delivery within its borders and chooses its own implementation strategy, results from one province cannot be extrapolated to the entire country. As mentioned, we have not identified other data on South Africa’s national or provincial TB treatment initiation trends with which to compare our results. We can, however, consider changes in HIV treatment initiations in South Africa during the same time period, that draws on the same data set and identical methodology [25]. Both HIV and TB are diagnosed mainly at primary health clinics, and COVID-19 may thus have affected treatment initiations for both diseases similarly. In both studies, facilities in rural areas were less affected than those in urban and peri urban areas. Differences in numbers of ART initiations and TB initiations were observed by facility level, however. Primary health clinics appeared to experience larger decreases in TB treatment initiations than in ART initiations, while hospitals had much smaller decreases in TB treatment initiations than in ART initiations. In both studies, treatment initiation numbers were generally inversely associated with COVID-19 caseload.

Our study had a number of limitations. First, as explained above, our full data set included only public sector health facilities in the DHIS that reported monthly observations in 2019, and our limited data set, which required that a facility report an observation in every month of the study period, represented only about 29% of the original DHIS data set. The selection bias introduced by excluding facilities with missing data is unclear, but we speculate that it may have biased the sample in favour of better-resourced and/or better-performing sites. In particular, as evident in S1 Table, there was a large concentration of missing data in the months of January, February, and March in both 2019 and 2020. We cannot explain this anomaly. It has the effect of diminishing the generalizability of results for those months, particularly in Limpopo Province, which was missing observations for up to 60% of its facilities in those months, and for rural facilities.

Second, we had no way to validate the data in the DHIS database, but as noted above, we know from others’ analyses that accuracy is variable. Third, our study period ended in April 2021, after the end of South Africa’s second wave of COVID-19. We do not know if TB treatment initiation numbers recovered at all prior to the start of the third wave in June 2021 or what has happened to the TB cascade of care since then. Fourth, our comparison values, from 2019, may or may not be a valid counter-factual for 2020 and early 2021. TB incidence in South Africa has been decreasing over the last decade, leading to a small decrease in the number of patients eligible for TB treatment initiation each year [21]. On the other hand, efforts made as part of the 2017–2022 national strategic plan may have led to improved case finding in 2020, in the absence of the pandemic.

As implied above, our data cannot tell us whether the observed reduction in initiations reflects COVID-19 morbidity, fear of COVID-19 transmission, reduction in the health system’s capacity to provide services, reallocation of healthcare resources to manage COVID-19 cases, constraints on movement due to the lockdown, or even reduced TB transmission due to COVID-19 related behaviour changes. We believe that it was almost certainly a combination of several of these factors, the same conclusion drawn in a global analysis [13]. The timing of the trends in TB treatment initiations suggests that less impact can be attributed to official lockdowns, and more to the pandemic itself, than may be commonly thought, though the role played by the large dropoff in TB testing, noted above, is unclear.

Regardless of the causes, South Africa’s progress toward reducing the impacts of TB in the country took a clear blow during the pandemic, and exceptional efforts and investment will be needed to recover that progress going forward [10], in South Africa and in other high TB burden countries. South Africa is privileged to have more complete data for those accessing health care in the public sector than do most of its neighbors, and data from South Africa may provide insights into the potential impact of COVID-19 in countries where national data are not available. It will be of utmost important that researchers continue to analyze DHIS data in the months since our data set ended, to deepen understanding of this issue.

## Supporting information

S1 Checklist Inclusivity in global research questionnaire. (DOCX)

S1 Table Number of facilities included in and excluded from the full data set in each month in the observation period. (DOCX)
