# Supplementary material for: Reduction in initiations of drug-sensitive tuberculosis treatment in South Africa during the COVID-19 pandemic: Analysis of retrospective, facility-level data
Source: PLOS Glob Public Health. 2022 Oct 11;2(10):e0000559. doi: 10.1371/journal.pgph.0000559 (PMC10021649; doi:10.1371/journal.pgph.0000559)
Supplement: S1 Table — (DOCX) [file pgph.0000559.s002.docx]

|  |  | January | | | | | | February | | | | | | March | | | | | | April | | | | | |
| --- | --- | --- | --- | --- | --- | --- | --- | --- | --- | --- | --- | --- | --- | --- | --- | --- | --- | --- | --- | --- | --- | --- | --- | --- | --- |
|  |  | 2019 | | 2020 | | 2021 | | 2019 | | 2020 | | 2021 | | 2019 | | 2020 | | 2021 | | 2019 | | 2020 | | 2021 | |
|  |  | Incl | Incl % | Incl | Incl % | Incl | Incl % | Incl | Incl % | Incl | Incl % | Incl | Incl % | Incl | Incl % | Incl | Incl % | Incl | Incl % | Incl | Incl % | Incl | Incl % | Incl | Incl % |
| Province |  |  |  |  |  |  |  |  |  |  |  |  |  |  |  |  |  |  |  |  |  |  |  |  |  |
| Eastern Cape | 890 | 630 | 70.8% | 623 | 70.0% | 878 | 98.7% | 604 | 67.9% | 568 | 63.8% | 878 | 98.7% | 606 | 68.1% | 593 | 66.6% | 876 | 98.4% | 889 | 99.9% | 879 | 98.8% | 877 | 98.5% |
| Free State | 285 | 192 | 67.4% | 195 | 68.4% | 281 | 98.6% | 171 | 60.0% | 186 | 65.3% | 279 | 97.9% | 171 | 60.0% | 179 | 62.8% | 280 | 98.2% | 281 | 98.6% | 282 | 98.9% | 270 | 94.7% |
| Gauteng | 423 | 332 | 78.5% | 347 | 82.0% | 407 | 96.2% | 331 | 78.3% | 328 | 77.5% | 406 | 96.0% | 321 | 75.9% | 328 | 77.5% | 405 | 95.7% | 419 | 99.1% | 418 | 98.8% | 411 | 97.2% |
| KwaZulu-Natal | 786 | 536 | 68.2% | 543 | 69.1% | 777 | 98.9% | 527 | 67.0% | 513 | 65.3% | 778 | 99.0% | 530 | 67.4% | 502 | 63.9% | 778 | 99.0% | 774 | 98.5% | 780 | 99.2% | 764 | 97.2% |
| Limpopo | 497 | 242 | 48.7% | 225 | 45.3% | 464 | 93.4% | 200 | 40.2% | 205 | 41.2% | 459 | 92.4% | 199 | 40.0% | 215 | 43.3% | 458 | 92.2% | 489 | 98.4% | 461 | 92.8% | 463 | 93.2% |
| Mpumalanga | 321 | 200 | 62.3% | 196 | 61.1% | 311 | 96.9% | 169 | 52.6% | 188 | 58.6% | 313 | 97.5% | 186 | 57.9% | 205 | 63.9% | 313 | 97.5% | 305 | 95.0% | 319 | 99.4% | 307 | 95.6% |
| North West | 341 | 213 | 62.5% | 226 | 66.3% | 329 | 96.5% | 199 | 58.4% | 202 | 59.2% | 326 | 95.6% | 180 | 52.8% | 203 | 59.5% | 330 | 96.8% | 340 | 99.7% | 336 | 98.5% | 325 | 95.3% |
| Northern Cape | 204 | 120 | 58.8% | 126 | 61.8% | 194 | 95.1% | 122 | 59.8% | 114 | 55.9% | 192 | 94.1% | 113 | 55.4% | 107 | 52.5% | 191 | 93.6% | 202 | 99.0% | 196 | 96.1% | 190 | 93.1% |
| Western Cape | 386 | 284 | 73.6% | 293 | 75.9% | 386 | 100.0% | 286 | 74.1% | 298 | 77.2% | 385 | 99.7% | 286 | 74.1% | 298 | 77.2% | 384 | 99.5% | 384 | 99.5% | 386 | 100.0% | 384 | 99.5% |
| Setting |  |  |  |  |  |  |  |  |  |  |  |  |  |  |  |  |  |  |  |  |  |  |  |  |  |
| Peri-Urban | 405 | 260 | 64.2% | 283 | 69.9% | 395 | 97.5% | 259 | 64.0% | 261 | 64.4% | 394 | 97.3% | 250 | 61.7% | 252 | 62.2% | 393 | 97.0% | 402 | 99.3% | 399 | 98.5% | 388 | 95.8% |
| Rural | 1912 | 1,077 | 56.3% | 1,053 | 55.1% | 1,869 | 97.8% | 991 | 51.8% | 951 | 49.7% | 1,867 | 97.6% | 996 | 52.1% | 1,004 | 52.5% | 1,867 | 97.6% | 1,887 | 98.7% | 1,867 | 97.6% | 1,860 | 97.3% |
| Urban | 1816 | 1,412 | 77.8% | 1,438 | 79.2% | 1,763 | 97.1% | 1,359 | 74.8% | 1,390 | 76.5% | 1,755 | 96.6% | 1,346 | 74.1% | 1,374 | 75.7% | 1,755 | 96.6% | 1,794 | 98.8% | 1,791 | 98.6% | 1,743 | 96.0% |
| Facility Type |  |  |  |  |  |  |  |  |  |  |  |  |  |  |  |  |  |  |  |  |  |  |  |  |  |
| Clinic | 3044 | 2,110 | 69.3% | 2,099 | 69.0% | 3,018 | 99.1% | 1,976 | 64.9% | 1,929 | 63.4% | 3,013 | 99.0% | 1,953 | 64.2% | 1,972 | 64.8% | 3,009 | 98.9% | 3,019 | 99.2% | 3,018 | 99.1% | 2,992 | 98.3% |
| Community centres | 332 | 288 | 86.7% | 282 | 84.9% | 329 | 99.1% | 263 | 79.2% | 281 | 84.6% | 327 | 98.5% | 265 | 79.8% | 273 | 82.2% | 325 | 97.9% | 330 | 99.4% | 331 | 99.7% | 326 | 98.2% |
| Hospitals | 293 | 235 | 80.2% | 269 | 91.8% | 289 | 98.6% | 235 | 80.2% | 260 | 88.7% | 288 | 98.3% | 240 | 81.9% | 251 | 85.7% | 287 | 98.0% | 289 | 98.6% | 291 | 99.3% | 281 | 95.9% |
| Others | 464 | 116 | 25.0% | 124 | 26.7% | 391 | 84.3% | 135 | 29.1% | 132 | 28.4% | 388 | 83.6% | 134 | 28.9% | 134 | 28.9% | 394 | 84.9% | 445 | 95.9% | 417 | 89.9% | 392 | 84.5% |
| **Total** | **4133** | **2749** | **66.5%** | **2774** | **67.1%** | **4027** | **97.4%** | **2609** | **63.1%** | **2602** | **63.0%** | **4016** | **97.2%** | **2592** | **62.7%** | **2630** | **63.6%** | **4,015** | **97.1%** | **4083** | **98.8%** | **4057** | **98.2%** | **3991** | **96.6%** |

**Supplemental table 1. Number of facilities included in and excluded from the full data set in each month in the observation period**

|  |  | May | | | | June | | | | July | | | | August | | | | September | | | |
| --- | --- | --- | --- | --- | --- | --- | --- | --- | --- | --- | --- | --- | --- | --- | --- | --- | --- | --- | --- | --- | --- |
|  |  | 2019 | | 2020 | | 2019 | | 2020 | | 2019 | | 2020 | | 2019 | | 2020 | | 2019 | | 2020 | |
|  |  | Incl | Incl % | Incl | Incl % | Incl | Incl % | Incl | Incl % | Incl | Incl % | Incl | Incl % | Incl | Incl % | Incl | Incl % | Incl | Incl % | Incl | Incl % |
| Province |  |  |  |  |  |  |  |  |  |  |  |  |  |  |  |  |  |  |  |  |  |
| Eastern Cape | 890 | 889 | 99.9% | 876 | 98.4% | 889 | 99.9% | 876 | 98.4% | 886 | 99.6% | 877 | 98.5% | 889 | 99.9% | 878 | 98.7% | 889 | 99.9% | 878 | 98.7% |
| Free State | 285 | 283 | 99.3% | 280 | 98.2% | 282 | 98.9% | 280 | 98.2% | 281 | 98.6% | 280 | 98.2% | 282 | 98.9% | 280 | 1.2% | 280 | 98.2% | 281 | 98.6% |
| Gauteng | 423 | 418 | 98.8% | 409 | 96.7% | 420 | 99.3% | 407 | 96.2% | 420 | 99.3% | 392 | 92.7% | 420 | 99.3% | 405 | 0.8% | 416 | 98.3% | 402 | 95.0% |
| KwaZulu-Natal | 786 | 776 | 98.7% | 775 | 98.6% | 775 | 98.6% | 777 | 98.9% | 784 | 99.7% | 781 | 99.4% | 784 | 99.7% | 780 | 0.5% | 785 | 99.9% | 781 | 99.4% |
| Limpopo | 497 | 490 | 98.6% | 455 | 91.5% | 491 | 98.8% | 460 | 92.6% | 492 | 99.0% | 459 | 92.4% | 482 | 97.0% | 460 | 4.7% | 494 | 99.4% | 461 | 92.8% |
| Mpumalanga | 321 | 317 | 98.8% | 312 | 97.2% | 310 | 96.6% | 314 | 97.8% | 317 | 98.8% | 310 | 96.6% | 315 | 98.1% | 311 | 4.2% | 312 | 97.2% | 311 | 96.9% |
| North West | 341 | 337 | 98.8% | 331 | 97.1% | 338 | 99.1% | 331 | 97.1% | 339 | 99.4% | 329 | 96.5% | 337 | 98.8% | 320 | 4.9% | 340 | 99.7% | 324 | 95.0% |
| Northern Cape | 204 | 203 | 99.5% | 193 | 94.6% | 203 | 99.5% | 187 | 91.7% | 203 | 99.5% | 190 | 93.1% | 203 | 99.5% | 192 | 3.1% | 203 | 99.5% | 194 | 95.1% |
| Western Cape | 386 | 384 | 99.5% | 386 | 100.0% | 384 | 99.5% | 386 | 100.0% | 385 | 99.7% | 386 | 100.0% | 385 | 99.7% | 386 | 0.4% | 385 | 99.7% | 386 | 100.0% |
| Setting |  |  |  |  |  |  |  |  |  |  |  |  |  |  |  |  |  |  |  |  |  |
| Peri-Urban | 405 | 402 | 99.3% | 393 | 97.0% | 402 | 99.3% | 398 | 98.3% | 404 | 99.8% | 394 | 97.3% | 403 | 99.5% | 396 | 97.8% | 405 | 100.0% | 396 | 97.8% |
| Rural | 1912 | 1,894 | 99.1% | 1,856 | 97.1% | 1,893 | 99.0% | 1,862 | 97.4% | 1,902 | 99.5% | 1,864 | 97.5% | 1,891 | 98.9% | 1,859 | 97.2% | 1,902 | 99.5% | 1,862 | 97.4% |
| Urban | 1816 | 1,801 | 99.2% | 1,768 | 97.4% | 1,797 | 99.0% | 1,758 | 96.8% | 1,801 | 99.2% | 1,746 | 96.1% | 1,803 | 99.3% | 1,757 | 96.8% | 1,797 | 99.0% | 1,760 | 96.9% |
| Facility Type |  |  |  |  |  |  |  |  |  |  |  |  |  |  |  |  |  |  |  |  |  |
| Clinic | 3044 | 3,030 | 99.5% | 3,002 | 98.6% | 3,023 | 99.3% | 3,002 | 98.6% | 3,031 | 99.6% | 2,992 | 98.3% | 3,017 | 99.1% | 3,005 | 98.7% | 3,030 | 99.5% | 3,011 | 98.9% |
| Community Centres | 332 | 328 | 98.8% | 328 | 98.8% | 330 | 99.4% | 329 | 99.1% | 329 | 99.1% | 325 | 97.9% | 328 | 98.8% | 327 | 98.5% | 330 | 99.4% | 327 | 98.5% |
| Hospitals | 293 | 291 | 99.3% | 288 | 98.3% | 291 | 99.3% | 287 | 98.0% | 290 | 99.0% | 289 | 98.6% | 293 | 100.0% | 291 | 99.3% | 290 | 99.0% | 290 | 99.0% |
| Others | 464 | 448 | 96.6% | 399 | 86.0% | 448 | 96.6% | 400 | 86.2% | 457 | 98.5% | 398 | 85.8% | 459 | 98.9% | 389 | 83.8% | 454 | 97.8% | 390 | 84.1% |
| **Total** | **4133** | **4097** | **99.1%** | **4017** | **97.2%** | **4092** | **99.0%** | **4018** | **97.2%** | **4107** | **99.4%** | **4004** | **96.9%** | **4097** | **99.1%** | **4012** | **97.1%** | **4104** | **99.3%** | **4018** | **97.2%** |

|  |  | October | | | | November | | | | December | | | |
| --- | --- | --- | --- | --- | --- | --- | --- | --- | --- | --- | --- | --- | --- |
|  |  | 2019 | | 2020 | | 2019 | | 2020 | | 2019 | | 2020 | |
|  |  | Incl | Incl % | Incl | Incl % | Incl | Incl % | Incl | Incl % | Incl | Incl % | Incl | Incl % |
| Province |  |  |  |  |  |  |  |  |  |  |  |  |  |
| Eastern Cape | 890 | 890 | 100.0% | 878 | 98.7% | 889 | 99.9% | 878 | 98.7% | 890 | 100.0% | 877 | 98.5% |
| Free State | 285 | 284 | 99.6% | 281 | 98.6% | 281 | 98.6% | 280 | 98.2% | 281 | 98.6% | 280 | 98.2% |
| Gauteng | 423 | 418 | 98.8% | 409 | 96.7% | 419 | 99.1% | 405 | 95.7% | 416 | 98.3% | 404 | 95.5% |
| KwaZulu-Natal | 786 | 783 | 99.6% | 781 | 99.4% | 782 | 99.5% | 780 | 99.2% | 785 | 99.9% | 781 | 99.4% |
| Limpopo | 497 | 494 | 99.4% | 461 | 92.8% | 496 | 99.8% | 461 | 92.8% | 495 | 99.6% | 461 | 92.8% |
| Mpumalanga | 321 | 316 | 98.4% | 311 | 96.9% | 316 | 98.4% | 308 | 96.0% | 311 | 96.9% | 309 | 96.3% |
| North West | 341 | 338 | 99.1% | 325 | 95.3% | 338 | 99.1% | 324 | 95.0% | 337 | 98.8% | 328 | 96.2% |
| Northern Cape | 204 | 203 | 99.5% | 194 | 95.1% | 203 | 99.5% | 194 | 95.1% | 202 | 99.0% | 192 | 94.1% |
| Western Cape | 386 | 386 | 100.0% | 386 | 100.0% | 386 | 100.0% | 386 | 100.0% | 386 | 100.0% | 386 | 100.0% |
| Setting |  |  |  |  |  |  |  |  |  |  |  |  |  |
| Peri-Urban | 405 | 403 | 99.5% | 397 | 98.0% | 402 | 99.3% | 398 | 98.3% | 403 | 99.5% | 398 | 98.3% |
| Rural | 1912 | 1,903 | 99.5% | 1,862 | 97.4% | 1,906 | 99.7% | 1,859 | 97.2% | 1,902 | 99.5% | 1,860 | 97.3% |
| Urban | 1816 | 1,806 | 99.4% | 1,767 | 97.3% | 1,802 | 99.2% | 1,759 | 96.9% | 1,798 | 99.0% | 1,760 | 96.9% |
| Facility Type |  |  |  |  |  |  |  |  |  |  |  |  |  |
| Clinic | 3044 | 3,032 | 99.6% | 3,012 | 98.9% | 3,036 | 99.7% | 3,008 | 98.8% | 3,031 | 99.6% | 3,015 | 99.0% |
| Community Centres | 332 | 329 | 99.1% | 326 | 98.2% | 331 | 99.7% | 326 | 98.2% | 328 | 98.8% | 325 | 97.9% |
| Hospitals | 293 | 293 | 100.0% | 290 | 99.0% | 289 | 98.6% | 289 | 98.6% | 290 | 99.0% | 286 | 97.6% |
| Others | 464 | 458 | 98.7% | 398 | 85.8% | 454 | 97.8% | 393 | 84.7% | 454 | 97.8% | 392 | 84.5% |
| **Total** | **4133** | **4112** | **99.5%** | **4026** | **97.4%** | **4110** | **99.4%** | **4016** | **97.2%** | **4103** | **99.3%** | **4018** | **97.2%** |
